# Multiphasic Evidential Decision-Making Matrix (MedMax) for Intrahepatic Cholangiocarcinoma: A Single-Center Validation Study

**DOI:** 10.3390/cancers18091365

**Published:** 2026-04-24

**Authors:** Ali Ramouz, Ali Adeliansedehi, Behboud Moeini Chagervand, Nastaran Sabetkish, Benjamin Goeppert, Christoph Springfeld, Elias Khajeh, Arianeb Mehrabi, Ali Majlesara

**Affiliations:** 1 Department of General, Visceral, and Transplantation Surgery, University Hospital Heidelberg, 69120 Heidelberg, Germany; ali.ramouz@med.uni-heidelberg.de (A.R.);; 2 Institute of Pathology, RKH Klinikum, 71640 Ludwigsburg, Germany; 3 Department of Medical Oncology, University Hospital Heidelberg, 69120 Heidelberg, Germany; 4 Liver Cancer Center Heidelberg, University Hospital Heidelberg, 69120 Heidelberg, Germany

**Keywords:** intrahepatic cholangiocarcinoma, decision matrix, MedMax, surgical decision-making, individualized therapy, retrospective validation

## Abstract

Background: Intrahepatic cholangiocarcinoma (ihCC) is a rare aggressive liver malignancy with rising incidence. For resectable cases, surgery is the only curative approach, but recurrence rates remain high. These challenges highlight the need for personalized, evidence-based clinical decision-making to improve patient outcomes. To address this, we developed the Multiphasic Evidential Decision-making Matrix (MedMax) to support systematic, individualized therapeutic strategies for ihCC. Methods: In this retrospective single-center study, between 2010 and 2020, we assessed the ability of the MedMax matrix to make treatment decisions in 489 consecutive patients with ihCC or suspected ihCC. Patients were divided into two cohorts depending on whether their tumor was operable (surgical cohort, n = 335) or non-operable (non-surgical cohort, n = 154). We assessed the accuracy of diagnostic confirmation and treatment allocation by MedMax and evaluated how the model’s recommendations corresponded to those made by the tumor board. Results: In the surgical cohort, MedMax achieved 100% accuracy in diagnostic confirmation and 97.9% accuracy in treatment allocation. There was 74.3% concordance between the resection type proposed by MedMax and actual extent of resection. This discrepancy was caused by deviations from the preoperative plan based on intraoperative findings, which could not have been predicted preoperatively. In the non-surgical cohort, MedMax again achieved 100% accuracy in diagnostic confirmation and 98.7% accuracy in treatment allocation. All discrepancies between the decisions made by MedMax and those made by the tumor board were attributed complex, high-risk patient profiles. MedMax reliably identified risk factors (such as advanced comorbidities and multifocal disease) in both cohorts. Conclusions: The MedMax matrix can make accurate, reliable and transparent decisions about the diagnosis and treatment of patients with ihCC thanks to its modular, evidence-based approach. It can also stratify and document risks in both surgical and non-surgical settings.

## Simple Summary

This study evaluated the MedMax decision-making matrix for managing intrahepatic cholangiocarcinoma in 489 patients. The system showed 100% accuracy in diagnosis and ~98% accuracy in treatment allocation in both surgical and non-surgical cases. Discrepancies with clinical decisions mainly resulted from unexpected intraoperative findings or complex patient risk profiles, indicating MedMax is a reliable tool for evidence-based treatment planning.

## 1. Introduction

Intrahepatic cholangiocarcinoma (ihCC) is a rare and highly aggressive malignancy of the biliary tract that arises predominantly in the segmental bile ducts [1,2,3]. The incidence and mortality of ihCC have increased over the past four decades and are expected to increase further, particularly among elderly males [4,5,6]. Despite advances in diagnostic and therapeutic strategies, the prognosis of patients with intrahepatic cholangiocarcinoma remains limited. Population-based analyses report overall 5-year survival rates of approximately 10% across all stages, with median overall survival ranging from about 8 to 12 months in unselected cohorts [7]. Outcomes vary substantially according to treatment setting and tumor biology. In patients undergoing curative-intent resection, median overall survival typically ranges from approximately 27 to 46 months, with 5-year survival rates between 25% and 44% [8,9,10], whereas patients with unresectable or metastatic disease receiving systemic therapy generally achieve median overall survival of approximately 11 to 13 months under current first-line regimens [3,11]. This poor prognosis is largely caused by the insidious onset and non-specific symptoms of the disease, which delay diagnosis [3,12]. Typically, patients are not diagnosed until the disease is metastatic or unresectable, at which point effective systemic therapies are scarce [3,13]. The only curative treatment for ihCC is surgical resection of the tumor with R0 margins and systematic lymphadenectomy, but this is only possible in patients with localized, resectable tumors [14,15,16]. In addition, tumor recurrence occurs within five years of hepatectomy in over 60% of patients [17,18]. This underscores the urgent need for personalized and evidence-based preoperative decision-making to identify optimal therapeutic strategies for patients with ihCC [3,8].

To address this problem, we previously developed and prospectively validated the Multiphasic Evidential Decision-making Matrix (MedMax) [19]. This matrix uses established decision-making pathways and incorporates expert opinions from tumor board discussions to evaluate diagnosis and treatment options and to determine whether patients are eligible for surgery. In a prospective analysis of 44 patients undergoing surgery for ihCC, we showed that MedMax was able to diagnose ihCC and select patients for surgery with 100% accuracy, and that the extent of resection proposed by MedMax showed 77.3% concordance with the actual extent of resection. In this retrospective analysis, we further validate the clinical relevance of the MedMax matrix by investigating its applicability and reliability in both operable and non-operable patients.

## 2. Materials and Methods

### 2.1. The MedMax Matrix

In this study, we evaluated the ability of the MedMax matrix to predict the optimal treatment and outcome in patients who underwent treatment for ihCC at Heidelberg University Hospital. The preoperative decision-making process of MedMax has been described in detail before [19] and consists of three phases: confirmation of diagnosis, definition of treatment (i.e., surgery or non-surgery), and planning of surgical treatment [19].

### 2.2. Study Cohort

In this retrospective single-center study, we evaluated the ability of the MedMax decision-making matrix to support diagnostic confirmation and treatment allocation in consecutive adult patients with ihCC or suspected ihCC, between 2010 and 2020. Inclusion criteria comprised patients undergoing primary surgical or non-surgical treatment for intrahepatic cholangiocarcinoma, while patients requiring emergency surgery were excluded. MedMax decisions were based on predefined clinical, laboratory, imaging, and histological parameters. Non-surgical therapy comprised neoadjuvant or palliative systemic therapy. The primary endpoints were concordance between MedMax recommendations and multidisciplinary tumor board decisions for diagnostic classification and treatment allocation. The need for ethical approval to collect patient data was waived by the Heidelberg University Hospital ethics committee because of the retrospective nature of the study (approval number: S-754/2018). The data were derived from a prospectively maintained patient database that contained information on adult patients not requiring emergency surgery.

### 2.3. Variables and Data Extraction

We extracted data on 45 parameters, and MedMax based its decisions on these parameters. These included baseline characteristics, medical history, preoperative diagnostic assessments, tumor resectability, and surgical planning. We also collected demographic and clinical factors, preoperative laboratory parameters, comorbidities, preoperative histopathological sampling, and tumor- and surgery-related variables. A complete list of these variables can be found in our previous paper [19]. Preoperative histopathological sampling was incorporated into the MedMax decision-making matrix as a binary diagnostic variable indicating histological confirmation or exclusion of intrahepatic cholangiocarcinoma. The presence of histological confirmation was considered supportive evidence for diagnostic confirmation during the first phase of the MedMax workflow. In cases where histology was unavailable or inconclusive, diagnostic confirmation relied on concordant clinical and radiological findings according to multidisciplinary tumor board standards. No additional pathology-derived parameters, such as tumor grading, immunohistochemistry, cytology subtyping, or molecular markers, were included in the decision algorithm. Histopathological confirmation was not used for treatment allocation, risk stratification, or surgical planning but served solely as a diagnostic confirmation criterion within the structured rule-based framework.

Final histopathological findings were not incorporated into the MedMax decision-making algorithm, as these data become available only after surgical treatment. These variables are presented here to provide clinical context for the study population and to facilitate interpretation of treatment decisions.

### 2.4. Comparison of Decisions Made by MedMax and the Tumor Board

We evaluated the concordance between decisions made by MedMax and those made by the multidisciplinary tumor board in our department to determine whether MedMax can provide reliable decision support in routine clinical practice. To do this, we compared the recommendations made by MedMax with those made by the tumor board. We also considered the intraoperative decisions that affected treatment in those patients who underwent surgical treatment.

### 2.5. Statistical Analysis

Categorical data are presented as frequencies and proportions, and continuous data are presented as medians and interquartile ranges (IQR). We compared the treatment recommended by MedMax with that recommended by the tumor board and evaluated the agreement between the two. Descriptive agreement was assessed using a confusion matrix, and overall accuracy was calculated using exact binomial methods and presented with corresponding 95% confidence intervals (CIs). Sensitivity and specificity were estimated by treating chemotherapy as the positive reference class, and positive and negative predictive values are also reported. Agreement beyond chance was quantified using Cohen’s kappa. To assess discriminative performance, we calculated the c-statistic, which is equivalent to the area under the receiver operating characteristic curve (ROC), with 95% CIs using the DeLong method. All analyses were conducted in R (R Foundation for Statistical Computing, Vienna, Austria, version 2025.9.2).

## 3. Results

### 3.1. Patient Characteristics

A total of 489 patients were included in the study. Of these, 335 underwent surgical treatment and 154 non-surgical treatment. Patient data collected before treatment and classified by cohort (surgical or non-surgical) are presented in Table 1. The median patient age was 63 years and 56.4% (276/489 patients) were male. Regarding comorbidities, end-stage renal disease was reported in 2.6% of patients, chronic obstructive pulmonary disease in 2% of patients, heart failure in 0.6% of patients, chronic hepatitis B in 4.7% of patients, chronic hepatitis C in 1.2% of patients, liver cirrhosis in 3.9% of patients, and cholangitis in 1.8% of patients. Diagnostic imaging revealed that 27.2% of patients had suspected distant metastases, and the tumor board recommended that 26.4% of patients undergo non-surgical management. In the remaining 73.6% of patients who underwent surgical intervention, major hepatectomy was the predominant approach (83.6% of cases), followed by minor anatomical resections (16.1% of cases), and non-anatomical resection (0.3% of cases).

### 3.2. Stepwise Decision-Making with MedMax

**Figure 1:**
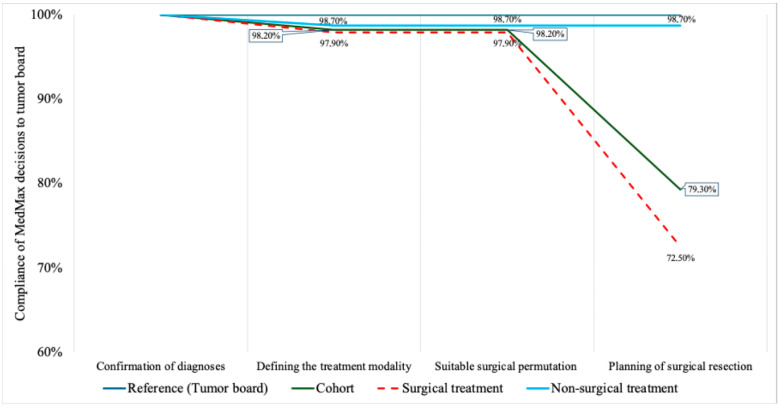
Compatibility between MedMax decisions and those made by the tumor board in patients with ihCC.

**Figure 2:**
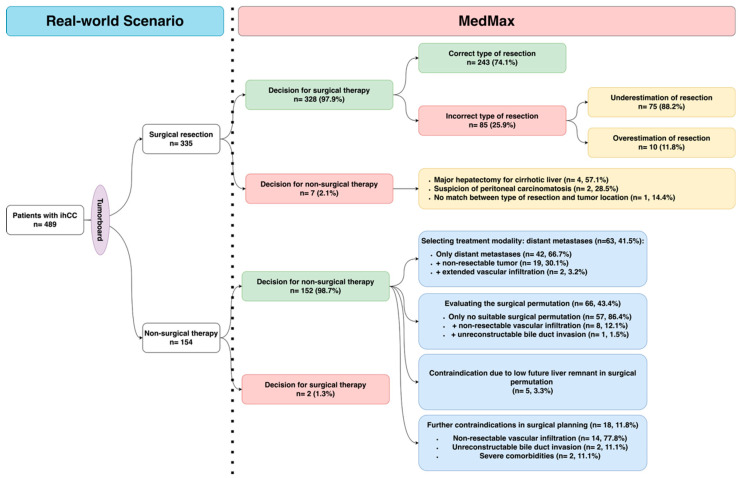
Flow chart of decisions made by MedMax after real-world decisions made by the tumor board.

The compatibility of the decisions made by MedMax with those made by the tumor board are shown in Figure 1. The first step of MedMax decision-making is diagnostic confirmation. MedMax classification of ihCC was fully concordant with the final clinical diagnosis in all cases (100% concordance). The second step of MedMax decision-making is treatment allocation. Here, the treatment recommendations made by MedMax were compatible with those made by the tumor board in 98.2% of patients (surgical treatment was recommended correctly in 97.9% of patients in the surgical cohort and non-surgical treatment was correctly recommended in 98.7% of patients in the non-surgical cohort). However, there were a few exceptions (Figure 2), which are detailed below.

#### 3.2.1. Disagreements Between MedMax and Tumor Board Decisions for Surgical Treatment

The tumor board allocated 335 patients to surgical resection, and MedMax recommended non-surgical therapy for seven of these patients (Figure 2). There were different reasons for these discrepancies between MedMax and tumor board decisions. In the first two patients, the matrix rejected surgery because it detected liver cirrhosis in one and suspicion of peritoneal carcinomatosis in the other based on imaging diagnostics. However, despite these contraindications, the tumor board decided that both patients should undergo exploratory laparotomy in our department, and peritoneal carcinomatosis was ruled out. In the third patient, the matrix found no suitable surgical approach for liver resection, so excluded the patient from surgery. However, the tumor board decided that this patient should undergo both anatomical and atypical liver resections for bilobar, multifocal intrahepatic cholangiocarcinoma. In the remaining four patients, the matrix excluded surgical resection because the patients had Child–Pugh class A cirrhosis; however, thanks to excellent perioperative management in our institution, these patients underwent the major hepatectomy, according to the tumor board decisions.

We also examined whether MedMax could help in surgical planning by accurately predicting the type of surgical treatment needed. The matrix correctly defined the type of surgical treatment in 243 of the 328 patients it allocated to surgical therapy (74.1% of patients). In the remaining 85/328 (25.9%) patients, the treatment predicted by MedMax was either misleading or differed from the procedure that was carried out. In 75/85 (88.2%) patients, MedMax suggested a less extensive resection than was performed, including mesohepatectomy instead of extended hemihepatectomy (39.2%), segmentectomy/bisegmentectomy instead of hemihepatectomy (33.5%), and hemihepatectomy instead of extended hemihepatectomy (15.4%). In the remaining 10/85 (11.8%) of patients, MedMax overestimated the extent of resection. It recommended segmentectomy/bisegmentectomy instead of wedge or atypical resection in 6.9% of patients and extended hemihepatectomy instead of hemihepatectomy with additional atypical resection in 4.9% of patients (Figure 2).

#### 3.2.2. Challenges of MedMax Decision-Making for Non-Surgical Treatment

The tumor board allocated 154 patients to non-surgical therapy, and MedMax recommended surgical resection in two of these patients (Figure 2). In the first patient, MedMax recommended an extended right hemihepatectomy because of the N status (multiple cN1 and cN2 lymph node metastases were confirmed histologically). However, CT imaging revealed a large lesion in the right liver lobe that partially infiltrated segment 4a, and the tumor board decided that surgical resection was oncologically unfeasible because of this extensive tumor infiltration and the multiple lymph node metastases. The patient received systemic therapy. In the second patient, MedMax recommended right hemihepatectomy despite numerous contraindications to surgery, including histologically confirmed ihCC in the right liver lobe with enlarged lymph nodes in stations 8, 12, and 15. Further risk factors were stage cN2, portal hypertension, ascites, portal vein thrombosis, and esophageal varices. The tumor board considered this cumulative risk too high for surgery and recommended systemic therapy.

MedMax correctly recommended non-surgical treatment in 152 patients. The main reason for rejecting surgery was lack of a suitable surgical approach for liver resection (43.4%), followed by distant metastases (41.5%), advanced tumors or comorbidities (11.8%), and low future liver remnant (3.3%). The full list of contraindications for surgical treatment defined by MedMax is presented in Figure 2.

### 3.3. Conformity of MedMax Decisions with Tumor Board Decisions

**Figure 3:**
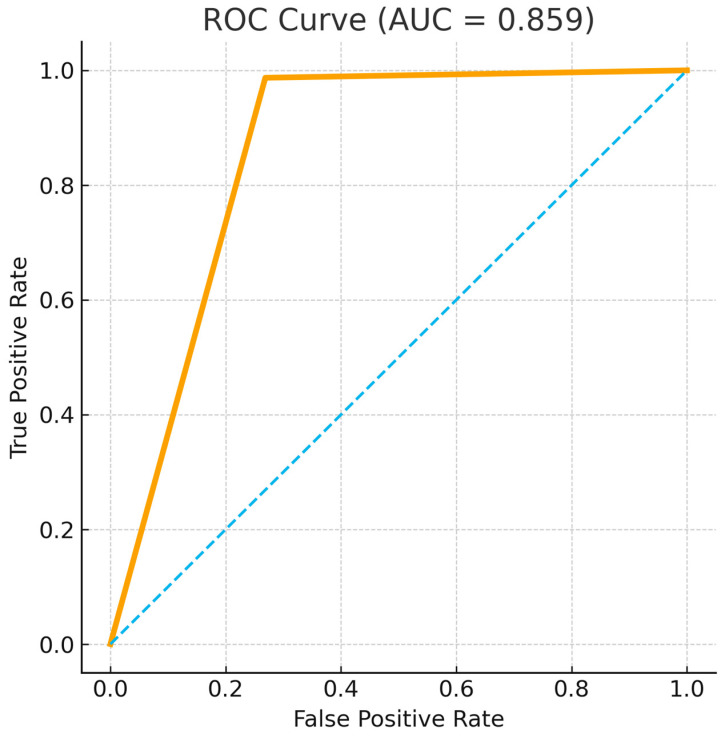
Ability of MedMax to differentiate between the need for surgical versus non-surgical therapy in patients with ihCC. The dashed diagonal line indicates the performance of a random classifier (AUC = 0.5), serving as a reference to assess the discriminative ability of the model.

The decisions made by MedMax conformed with those made by the tumor board in 81% of the cases (95% CI 77–85). MedMax recommendations for chemotherapy were almost always consistent with the tumor board decisions, reflected by a positive predictive value of 0.99 (95% CI, 0.95–1.00). MedMax also showed high specificity for identifying those patients that would most appropriately be managed with surgery (specificity, 0.99; 95% CI, 0.97–1.00) and rarely gave false positive recommendations for chemotherapy in these patients. However, MedMax showed more limited sensitivity to correctly identify patients requiring chemotherapy (sensitivity, 0.63; 95% CI, 0.56–0.69) and allocated some patients who needed chemotherapy for surgery (false negative rate, 0.37; 95% CI, 0.31–0.44). Overall, the ability of MedMax to distinguish between patients needing surgical and non-surgical treatment was good, with an area under the ROC curve of 0.86 (95% CI, 0.83–0.88) (Figure 3).

## 4. Discussion

In the present study, we validated the ability of MedMax to make treatment decisions in patients with resectable and non-resectable ihCC. We found that MedMax reliably identified appropriate candidates for surgical resection. MedMax also identified patients who needed chemotherapy with very high specificity, and this almost always aligned with the decision of the tumor board. This is clinically relevant because overtreatment with chemotherapy can have adverse outcomes in patients who would benefit more from tumor resection.

However, the matrix showed more limited sensitivity when identifying patients who needed chemotherapy, which meant that some patients who needed chemotherapy would have been allocated to undergo surgery by MedMax. Several factors may explain this low sensitivity. The first is that the tumor board decision is often influenced by nuanced clinical elements, such as board member experience, radiologic changes, tumor biology, liver function dynamics, performance status, prior treatment responses, and patient preference. These elements are not easily captured by a fixed rule-based matrix like MedMax. Another possible reason for the low sensitivity is that clinicians may have favored chemotherapy over surgery as a precaution in patients with borderline ihCC, whereas MedMax assigned treatment more rigidly. The low sensitivity of MedMax means that it may not fully account for clinical complexity in patients with more advanced or heterogeneous disease presentations.

A strength of MedMax is that its modular, machine-readable structure not only facilitates the construction of flexible, individualized clinical decision models but also lays the groundwork for future integration of artificial intelligence (AI) and progressive optimization (such as inclusion of prospective prognostic and recurrence models). AI and machine learning methodologies are being used to predict oncological outcomes [20,21], and these AI-driven systems can simultaneously integrate and analyze data from multiple sources, predict the risk of recurrence before resection [22,23,24], and determine optimal resection margins [22]. However, significant barriers remain, including model interpretability (“black box” challenge), the need for large multi-center datasets, and compliance with regulatory standards [25,26]. Explainable AI algorithms and continuous curation of structured databases are essential for sustainable clinical implementation. MedMax can easily implement predictive scores, biomarkers, and novel risk variables in a modular fashion, which means it can stratify patients far better than earlier, less adaptable systems could. This is relevant because patients with ihCC need more adaptive, patient-centered treatments, which means diverse clinical, molecular, and imaging-derived information needs to be integrated [27,28,29]. Iterative AI-assisted models that validate in real time and can adapt their learning capacities will be able to select individually tailored therapeutic interventions and dynamically respond to disease progression, which has not been possible so far [20,25,30]. As AI technologies advance and clinical data repositories expand, AI will likely become more useful in surgical oncology, particularly in identifying novel risk factors and in prospectively adapting therapeutic strategies [23,31].

Our findings show that MedMax can help with our clinical decision-making. Its high specificity indicates that it can effectively identify patients who are eligible for surgery and can standardize treatment planning in routine practice or in centers with limited multidisciplinary expertise. However, our finding that MedMax cannot identify patients who would benefit from chemotherapy with high sensitivity shows that it should not replace tumor board review completely, particularly in clinically complex or borderline cases. The sensitivity of MedMax may be improved by refining the matrix, for example by incorporating dynamic clinical variables, radiologic scoring systems, or molecular and biomarker data. Adapting the matrix based on machine learning using larger, multi-center datasets could also increase its ability to identify patients who would benefit most from chemotherapy. Taken together, our findings indicate that MedMax serves clinicians best as a structured aid within a broader multidisciplinary framework rather than as a standalone decision-making instrument.

There are some limitations to this study. First, the validation was retrospective and single-centered, and some operative decisions were heavily influenced by intraoperative findings that could not have been predicted preoperatively. Second, MedMax was limited in predicting the treatment and outcomes of certain cases, particularly atypical or extended bilobar lesions. Furthermore, the reported 100% rate of diagnostic confirmation should be interpreted cautiously. In this study, diagnostic confirmation reflects concordance between MedMax classification and the final clinical diagnosis rather than independent diagnostic accuracy. The study design did not evaluate the sensitivity or specificity of MedMax for detecting intrahepatic cholangiocarcinoma, and histological confirmation was not uniformly available prior to treatment in all cases. Therefore, this metric should be understood as a measure of decision consistency within a structured clinical workflow rather than diagnostic performance. Another limitation is that we cannot yet generalize these findings to other clinical settings or centers.

## 5. Conclusions

In summary, our analysis shows that MedMax is a promising, structured decision-support tool that can standardize and inform ihCC management but should complement—rather than replace—multidisciplinary clinical judgment pending prospective multicenter validation. The matrix provides a flexible, comprehensible, and precise strategy for selecting patients for either surgery or systemic therapy, minimizes the risk of oncologically inadequate interventions, and paves the way for the integration of AI-driven approaches into personalized oncologic care. Further multi-center studies are needed to confirm the external validity and clinical benefits of MedMax.

## Figures and Tables

**Table 1:** Patient data before treatment.

		N (%) or Median (IQR)	
		Surgical Therapy n = 335	Non-Surgical Therapy n = 154
**Demographic data**			
**Age**		63 (55–69)	63 (54–71)
**Gender**	*Female*	142 (42.4)	71 (46.1)
	*Male*	193 (57.6)	83 (53.9)
**BMI**		25.7 (23.1–28.6)	24.8 (23.2–27.1)
**Medical history**			
**Heart failure** **(NYHA classification)**	*I*	0 (0.0)	0 (0.0)
	*II*	2 (0.6)	0 (0.0)
	*III*	1 (0.3)	0 (0.0)
**Chronic obstructive pulmonary disease** **(GOLD classification)**	*I*	4 (40.0)	0 (0.0)
	*II*	4 (40.0)	0 (0.0)
	*III*	2 (20.0)	0 (0.0)
**Liver cirrhosis** **(Child–Pugh classification)**	*A*	11 (3.3)	8 (5.2)
	*B*	0 (0.0)	1 (0.6)
	*C*	0 (0.0)	0 (0.0)
**End-stage renal disease**		13 (3.9)	5 (3.2)
**Chronic hepatitis**	*B*	17 (5.1)	6 (3.9)
	*C*	4 (1.2)	2 (1.3)
**Cholangitis**		8 (2.4)	1 (0.6)
**Laboratory parameters**			
**Hemoglobin (g/dL)**		13.2 (12–14.1)	12.7 (11.2–13.8)
**Leukocytes (×10^9^/L)**		7.7 (6.2–9.4)	8.6 (6.4–10.8)
**Platelets (×10^9^/L)**		275 (213–347)	235 (156–311.5)
**C-reactive protein (mg/L)**		6.9 (2.2–14.4)	23.2 (10.5–57.1)
**Aspartate aminotransferase (U/L)**		33 (23–55)	57 (35–84)
**Alanine aminotransferase (U/L)**		32 (21–65.3)	36 (24–65)
**Total bilirubin (mg/dL)**		0.6 (0.4–1.1)	0.6 (0.4–1.4)
**Direct bilirubin (mg/dL)**		0.2 (0.1–9)	0.3 (0.2–1.4)
**Gamma-glutamyl transferase (U/L)**		106 (46–268.3)	335 (176.5–563.5)
**Alkaline phosphatase (U/L)**		126 (80.5–237)	228 (184–354)
**Prothrombin time (Quick, %)**		101 (91–109.5)	86.9 (70–103.4)
**Albumin (g/L)**		43.4 (40.7–46)	41.7 (36.9–44.4)
**Carcinoembryonic antigen (ng/mL)**		1.7 (0.9–3)	2.1 (1.2–7.2)
**Carbohydrate antigen 19-9 (U/mL)**		32.1 (13.7–174)	66.7 (20.7–1073.6)
**Alpha-1-fetoprotein (ng/mL)**		3.2 (1.8–6)	5.1 (2.5–14.1)
**Diagnostic outcomes**			
**Suspicion of distant metastases (X-ray, CT, MRI, EGD, colonoscopy)**		4 (1.1)	129 (83.8)
**Suspicion of regional lymph node metastases (CT, MRI)**		134 (40)	17 (11.0)
**Suspicion of peritoneal carcinomatosis (CT, MRI)**		2 (0.6)	16 (10.4)
**Portal hypertension (CT, MRI)**		2 (0.6)	1 (0.6)
**Ascites (ultrasound, CT)**	*A*	2 (0.6)	2 (1.3)
	*B*	1 (0.3)	0 (0.0)
	*C*	2 (0.6)	0 (0.0)

Abbreviations: BMI = body mass index, CT = computed tomography, EGD = esophagogastroduodenoscopy, GOLD = Global Initiative for Chronic Obstructive Lung Disease, IQR = interquartile range, MRI = magnetic resonance imaging, NYHA = New York Heart Association.

## Data Availability

The datasets generated and analyzed during the current study are available from the corresponding author on reasonable request.

## Abbreviations

The following abbreviations are used in this manuscript:

AI	Artificial intelligence
BMI	Body mass index
CI	Confidence interval
CT	Computer tomography
EGD	Esophagogastroduodenoscopy
GOLD	Global initiative for chronic obstructive lung disease
ihCC	Intrahepatic cholangiocarcinoma
IQR	Interquartile range
MedMax	Multiphasic Evidential Decision-making Matrix
MRI	Magnetic resonance imaging
NYHA	New York heart association
ROC	Receiver operating characteristic curve
